# Natural Disasters in the Americas, Dialysis Patients, and Implications for Emergency Planning: A Systematic Review

**DOI:** 10.5888/pcd17.190430

**Published:** 2020-06-11

**Authors:** Rashida S. Smith, Robert J. Zucker, Rosemary Frasso

**Affiliations:** 1 College of Population Health, Thomas Jefferson University, Philadelphia, Pennsylvania

## Abstract

**Introduction:**

Natural hazards are elements of the physical environment caused by forces extraneous to human intervention and may be harmful to human beings. Natural hazards, such as weather events, can lead to natural disasters, which are serious societal disruptions that can disrupt dialysis provision, a life-threatening event for dialysis-dependent people. The adverse outcomes associated with missed dialysis sessions are likely exacerbated in island settings, where health care resources and emergency procedures are limited. The effect of natural disasters on dialysis patients living in geographically vulnerable areas such as the Cayman Islands is largely understudied. To inform predisaster interventions, we systematically reviewed studies examining the effects of disasters on dialysis patients and discussed the implications for emergency preparedness in the Cayman Islands.

**Methods:**

Two reviewers independently screened 434 titles and abstracts from PubMed, Scopus, CINAHL, and Cochrane Library. We included studies if they were original research articles published in English from 2009 to 2019 and conducted in the Americas.

**Results:**

Our search yielded 15 relevant articles, which we included in the final analysis. Results showed that disasters have both direct and indirect effects on dialysis patients. Lack of electricity, clean water, and transportation, and closure of dialysis centers can disrupt dialysis care, lead to missed dialysis sessions, and increase the number of hospitalizations and use of the emergency department. Additionally, disasters can exacerbate depression and lead to posttraumatic stress disorder among dialysis patients.

**Conclusion:**

To our knowledge, this systematic review is the first study that presents a synthesis of the scientific literature on the effects of disasters on dialysis populations. The indirect and direct effects of disasters on dialysis patients highlight the need for predisaster interventions at the patient and health care system levels. Particularly, educating patients about an emergency renal diet and offering early dialysis can help to mitigate the negative effects of disasters.

**Summary**

**What is already known on this topic?**

Natural disasters can readily disrupt dialysis services, potentially resulting in hospitalizations and death among dialysis patients.

**What is added by this report?**

Disasters have direct and indirect effects on dialysis patients. Lack of electricity, clean water, and transportation, and closure of dialysis centers can disrupt dialysis care, lead to missed dialysis sessions, and increase the number of hospitalizations and use of the emergency department.

**What are the implications for public health practice?**

Mitigating the impacts of disasters on dialysis patients requires coordination among health professionals, carefully designed emergency preparedness plans, and education and training of all involved.

## Introduction

Natural hazards are elements of the physical environment that are caused by forces extraneous to human intervention and may be harmful to human beings. Natural hazards, such as weather events, can lead to natural disasters (hereinafter referred to as disasters), which are serious societal disruptions. Disasters can lead to disruption of dialysis provision, a life-threatening event for dialysis-dependent people. People with end-stage renal disease (ESRD) who are dialysis-dependent constitute a medically vulnerable population with high rates of health care use, morbidity, and mortality (1–3). Missed dialysis sessions exacerbate these adverse outcomes and correlate with a higher patient-perceived burden of kidney disease, higher mortality and hospitalization rates, increased emergency department (ED) visits, and worse general and mental health (4–7).

Disasters can affect access to dialysis by disrupting transportation, electricity, and water supply (8). Lack of transportation can leave dialysis patients immobile and unable to receive treatment. Similarly, loss of electricity and contamination of water systems can force dialysis centers to close, requiring dialysis patients to seek care elsewhere or miss treatments (9,10). The immediate threats from disasters are compounded by long-term stressors and mental health effects (11).

Just 577 miles south of Florida, the Cayman Islands is home to more than 68,000 people (12) and has more than 2 million visitors annually (13). As of 2018, the Cayman Islands had 4.1 physicians per 1,000 residents and fewer than 250 inpatient hospital beds (14,15). In addition to government health care services, the Cayman Islands have 100 private health care facilities (most of which are outpatient clinics) and 2 private hospitals; both hospitals are located on Grand Cayman, although neither provides dialysis services nor operates an ED (15). Hurricanes can disrupt dialysis provision, and dialysis patients may be flown overseas to receive care (16). However, patient transport is costly, and the dialysis population is growing; therefore, effective emergency preparedness programs are important in the Cayman Islands and other island settings.

The effect of disasters on dialysis patients living in geographically vulnerable areas such as the Cayman Islands is largely understudied. The objective of this systematic review was to describe the scope and effects of disasters on dialysis patients and the unique needs of dialysis patients during and after a disaster, to inform planning and effective emergency preparedness.

## Methods

### Data sources

From January 29, 2019, through February 1, 2019, we searched PubMed, Scopus, CINAHL, and the Cochrane Library to identify peer-reviewed studies published from January 1, 2009, through January 31, 2019, that reported on the effects of disasters on dialysis patients. We selected the search terms in consultation with a research librarian; they were a combination of Medical Subject Headings (Box) and keyword terms (full search string available in Appendix A). This review followed PRISMA (Preferred Reporting Items for Systematic Reviews and Meta-Analyses) guidelines (17).

**Box. Medical Subject Headings (MeSH) Search Terms Used in a Systematic Review of Natural Disasters in the Americas, Dialysis Patients, and Implications for Emergency Planning**

**Table:** 

Category	Search Terms
Problem	Disasters, natural disasters
Intervention	Kidney failure, dialysis
Outcomes	Delivery of health care, mortality, morbidity, hospitalization, emergency department use, adverse outcomes, health services accessibility, quality of life, patient satisfaction, patient care, patient experiences, patient care management, treatment outcome, mental health, complications, questionnaires and surveys

### Study selection

We reviewed studies that met the following inclusion criteria: they reported on the effects of disasters on dialysis patients; they were published in English from January 1, 2009, through January 31, 2019; and they were conducted in the Americas. We excluded review articles, editorials, and commentaries. However, we examined the reference sections of these articles for potentially relevant articles meeting our inclusion criteria.

For this review, “disasters” refer to all naturally occurring hazardous events of the physical environment such as hurricanes, tornadoes, and earthquakes, that can lead to human, material, economic, and/or environmental losses or impacts (18,19). The effects of disasters on patients can be direct or indirect. Direct effects include harm to the physical, mental, or social well-being of patients, and indirect effects include damage to health care facilities, dialysis centers, dialysis apparatus, water supply, electricity, or transportation.

Two authors (R.S.S. and R.J.Z.) performed independent reviews of the identified titles and abstracts to assess whether they met the inclusion criteria for full-text review. Next, these authors reviewed full-text articles and independently determined which articles to include for data extraction. They reviewed bibliographies to identify additional relevant articles and resolved discrepancies by consensus.

### Data extraction

Two reviewers (R.S.S. and R.J.Z.) independently extracted data from each study in the sample. They extracted the following information: author names, publication year, study objectives, study design, participant demographic characteristics, sample size, and relevant findings. We did not pre-identify outcome summary measures for data extraction because we considered multiple outcomes for inclusion. However, when a quantitative study reported an outcome of interest by using a summary measure, such as an odds ratio or hazard ratio, we extracted these data. Additionally, the 2 reviewers independently identified the direct and indirect effects of disasters on dialysis patients and categorized them as indirect effects, direct effects, mental health effects, and others.

Finally, the 2 reviewers independently assessed the quality of each study by using the following tools: the Newcastle–Ottawa Scale, a measurement tool for assessing the quality of observational cohort studies (20), the Critical Appraisal Skills Programme Qualitative Checklist (21), and the Joanna Briggs Checklist for Analytical Cross Sectional studies (22). Neither the Critical Appraisal Skills Programme Qualitative Checklist nor the Joanna Briggs checklist includes a scoring system. Therefore, the reviewers discussed and agreed on the overall value for qualitative studies and overall appraisal for cross-sectional studies. We converted the Newcastle–Ottawa Scale to good, fair, or poor quality categories by using a method described previously (23,24). The reviewers resolved discrepancies by consensus.

The study was not registered before data extraction, and the study design was developed in consultation with a research librarian.

## Results

The initial search yielded 434 articles published; we removed 56 duplicates and screened 378 titles and abstracts (Figure). After eliminating 357 articles that did not meet inclusion criteria, we assessed 21 full-text articles for eligibility. We excluded 2 review articles, and we removed 4 more articles that did not meet the inclusion criteria after closer review. Fifteen articles met the selection criteria for full-text data extraction (Table 1).

**Figure:**
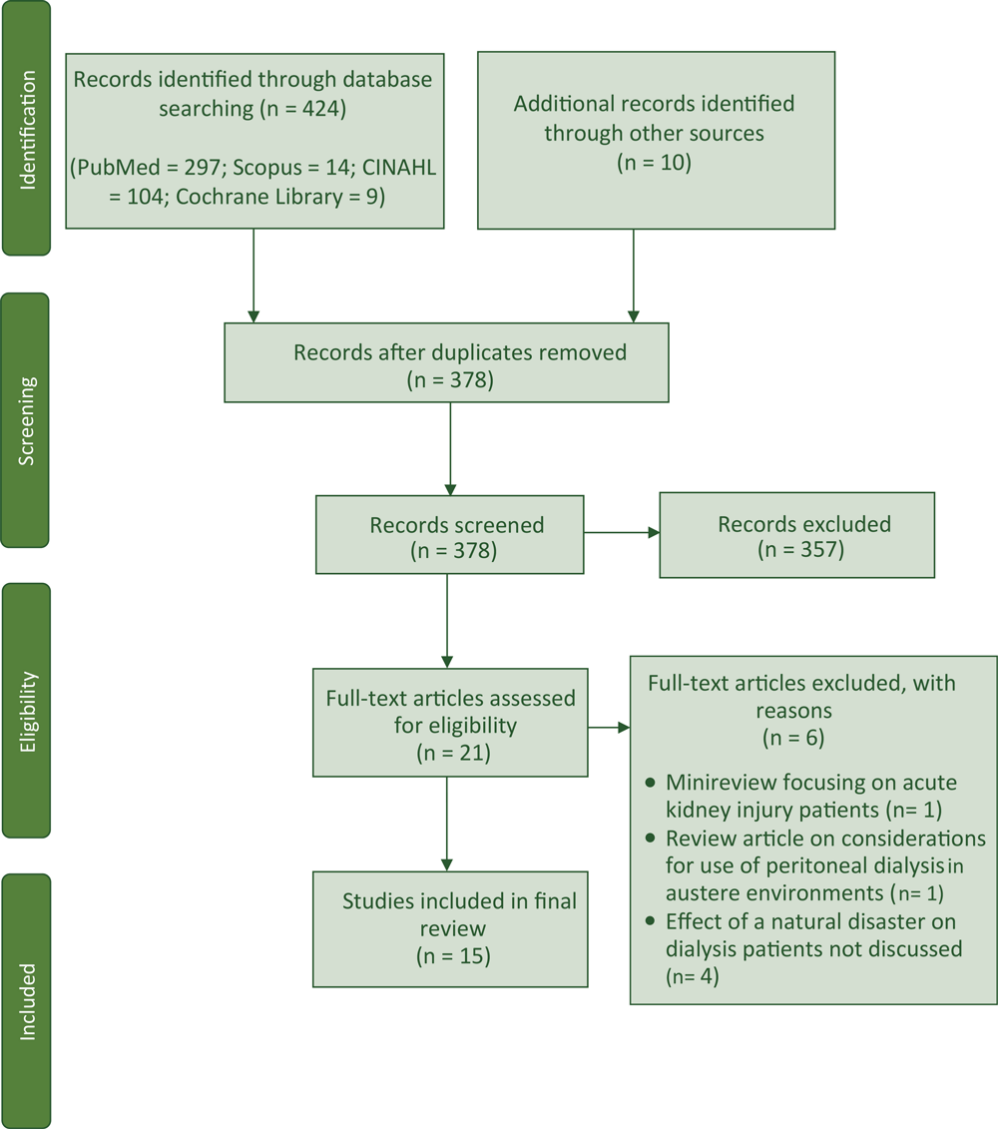
The study selection process for a systematic review of natural disasters in the Americas, dialysis patients, and implications for emergency planning. The search was conducted from January 29, 2019, through February 1, 2019.

**Table 1:** Results of Studies Reporting on the Effects of Natural Disasters in the Americas on Dialysis Patients, January 2009–January 2019

Authors	Study Location	Sample Characteristics and Size	Study Design	Study Objectives	Summary of Findings
**Hurricane Maria**					
Bonilla-Félix et al, 2018 (25)	San Juan, Puerto Rico	Pediatric patients with chronic renal disease; sample size not reported	Narrative report: personal recollections and experiences of the authors	Describe the authors’ experiences with patients with renal disease in an academic medical center	Shortage of fuel affected patient transportation services and personnel; peritoneal dialysis patients compensated by doing manual exchanges. Lack of electricity and potable water resulted in 3 cases of bacterial peritonitis; physicians forced to close their practices. Complete loss of the communication system resulted in difficulties sharing messages with patients about where to receive dialysis treatments; challenges communicating with dialysis centers and staff members. Blocked roads created challenges in moving patients.
**Hurricane Sandy**					
Malik et al, 2018 (26)	New York, New York	Adults aged 65 or older who used the ED post-disaster (N = 9,852 weekly average in the 43 weeks before Sandy; N = 10,073 average 1 week after Sandy)	Temporal and geospatial analysis; retrospective review of an all-payer claims database to analyze demographics, insurance status, geographic distribution and health conditions of older adults post-disaster	Evaluate the effect of Hurricane Sandy on ED use by older adults post-disaster, and characterize the primary and secondary medical needs of these people	Increase in overall average weekly ED visits for all evacuation zones in New York City in the first week after Hurricane Sandy. Greatest increase in ED use was by older adults in evacuation zone 1 (from 552 to 1,111; *P* < .01), the area most likely to flood. Significant increases (*P* < .05) in selected primary diagnoses among older adult ED patients in evacuation zone 1 in the week post-Sandy were found for dialysis (+1.9% among adults aged 65–74, +2.7% among adults aged 75–84, and +1.3% among adults aged ≥85); chronic kidney disease (+1.9% among adults aged 65–74 and +1.7% among adults aged 75–84); and electrolyte disorders (+1.9% among adults 75–84). Significant increases (*P* < .05) in selected secondary diagnoses among older adult ED patients in evacuation zone 1 in the week post-Sandy were dialysis (+1.4% among adults aged 65–74) and chronic kidney disease (+1.6% among adults aged 75–84 and +2.0% among adults aged ≥85).
Lee et al, 2016 (27)	New York, New York	Noninstitutionalized adult patients aged ≥18 who visited the ED in 2012 and had a home address in New York City (N = 50,996 one week before the hurricane; N = 46,131 one week after the hurricane)	Retrospective review of emergency claims data for adults visiting the ED in 2012; time-series analysis of frequency of visits for specific conditions and comorbidities	Characterize the geographic distribution of ED use post-Hurricane Sandy, and identify the post-disaster acute medical needs that developed in various geographic regions	From the day of the hurricane (day 0) through day 5, categories of primary ICD-9 diagnosis codes with significant (*P* < .001) increases among ED patients were chronic kidney disease, dialysis dependence, electrolyte abnormality, and renal failure. The significant increase in dialysis dependence lasted the longest of the 4 increases: it was significant (*P* < .001) from day 0 through day 5. The frequency of ED use significantly (*P* < .001) increased among patients with a secondary ICD-9 diagnosis category of dialysis dependence and chronic kidney disease.
Gotanda et al, 2015 (29)	Lower Manhattan, New York	Patients aged ≥18 who visited the Beth Israel Medical Center ED from May 7, 2012, through April 28, 2013 (n = 1,747 ED visits during the baseline phase; n = 1,766 ED visits during the immediate post-Sandy phase; n = 424 admissions during baseline phase; n = 516 admissions during the immediate post-Sandy phase)	Retrospective observational study using data from ED and hospital databases	Evaluate the impact of Hurricane Sandy on ED and hospital use for the geriatric population compared to adults aged <65 in lower Manhattan and determine the reasons for their ED visits and subsequent hospitalizations	Dialysis was 1 of the 4 concerns reported in EDs that significantly increased from baseline to the immediate post-Sandy phase (October 29–November 4, 2012) in all 3 age groups (18–64, 65–79, and ≥80; *P* < .05). Dialysis was 1 of the 3 chief reasons for hospital admission that significantly increased in all 3 age groups (*P* < .05). The largest increase was among adults aged ≥65. Dialysis peaked 2 days after the disaster.
Murakami et al, 2015 (30)	Lower Manhattan, New York	Patients aged ≥18 receiving dialysis care at 5 of 8 dialysis facilities in lower Manhattan at the time of Hurricane Sandy (n = 357)	Systematic cross-sectional 1-year follow-up survey: dialysis-specific preparedness was assessed by using the 13-item National Kidney Foundation–recommended dialysis-specific disaster preparedness checklist, and general disaster preparedness was assessed using the 15-item checklist proposed by the Department of Homeland Security	Describe the relationship between dialysis-specific and general disaster preparedness with missed dialysis sessions post-Sandy, for patients on hemodialysis	94 (26.3%) study participants missed dialysis; median number of dialysis sessions missed was 2 (interquartile range, 1–3). 65 (69.1%) participants missed 1 or 2 sessions, and 27 (28.7%) participants missed 3–5 sessions. Transportation (no. of study participants stating reason = 14/94; 14.9%), unit closure (n = 38/94; 40.4%), and both transportation and unit closure (n = 42/94; 44.7%) were cited as reasons for missing dialysis. 221 (61.9%) participants received early dialysis, and 57 (25.8%) of those that received early dialysis still missed dialysis sessions. Although early dialysis did not significantly change the number of participants missing dialysis (*P* = .81), it was associated with fewer missed dialysis sessions. 236 (66.1%) participants received dialysis at nonregular dialysis facilities; 209 (58.5% ) received dialysis at affiliated facilities, and 27 (7.6%) in EDs. Among those receiving dialysis at affiliated facilities or in EDs, 68 (28.8%) received shortened treatments, which led to overt symptoms in 11 participants. Several factors were associated with a significantly lower incidence of missed dialysis sessions after Hurricane Sandy: dialysis-specific preparedness (IRR, 0.91; 95% CI, 0.87–0.98; *P* = .001); a race/ethnicity other than white, black, or Hispanic (IRR, 0.34; 95% CI, 0.20–0.57; *P* < .001); dialysis treatment in an affiliated facility (IRR, 0.69; 95% CI, 0.51–0.94; *P* = .02); and older age (IRR, 0.98; 95% CI, 0.97–0.99; *P* < .001). Two factors were associated with a significantly higher number of missed dialysis sessions after Hurricane Sandy: the requirement for evacuation (IRR, 1.9; 95% CI, 1.1–2.3; *P* = .02) and a disturbed living situation (IRR, 2.3; 95% CI, 1.6–3.2; *P* < .001).
Lurie et al, 2015 (31)	New York, New York, and state of New Jersey	ESRD Medicare beneficiaries enrolled in Medicare Parts A and B receiving facility-based hemodialysis who had a claim for ≥1 maintenance dialysis treatment (from October 1 to October 28, 2012, in New York City and New Jersey) and were not hospitalized for the week of the storm (N = 13,836)	Retrospective cohort analysis using data from the Centers for Medicare & Medicaid Services Datalink Project	Examine the relationship between early dialysis and adverse outcomes (ie, ED visits, hospitalizations, and 30-day mortality after the storm) among patients with ESRD in the areas most affected by Sandy	8,256 (60%) study patients received early dialysis. In unadjusted analyses, patients receiving early dialysis had lower odds of ED visits (OR, 0.75; 95% CI, 0.63–0.89; *P* = .001) and hospitalization (OR, 0.77; 95% CI, 0.65–0.92; *P* = .004) than patients not receiving early dialysis.This pattern persisted in adjusted analyses; patients receiving early dialysis had lower odds than patients not receiving early dialysis of ED visits (OR, 0.80; 95% CI, 0.67–0.96; *P* = 0.01) and hospitalizations (OR, 0.79; 95% CI, 0.66–0.94; *P* = 0.01) in the week of the storm. In unadjusted analyses, the odds of 30-day mortality were similar among patients receiving early dialysis and patients not receiving early dialysis (OR, 0.80; 95% CI, 0.58–1.09; *P* = .20). However, in adjusted analyses early dialysis was significantly associated with reduced odds of 30-day mortality (OR, 0.72; 95% CI, 0.52–0.997; *P* = .048).
Kelman et al, 2015 (32)	New York, New York, and State of New Jersey	ESRD Medicare beneficiaries enrolled in Medicare Parts A and B receiving facility-based hemodialysis who had a claim for ≥1 maintenance dialysis treatment between October 1 and October 28, 2012, in New York City and the state of New Jersey (N = 13,264 study group patients)	Retrospective cohort study with 2 comparison groups using claims data from the Centers for Medicare & Medicaid Services Datalink Project. Study group consisted of ESRD patients in Sandy-affected areas. Comparison group 1 consisted of ESRD patients living in states unaffected by Sandy during the same period. Comparison group 2 consisted of ESRD patients living in the Sandy-affected region a year before the hurricane (October 1–October 30, 2011)	Characterize patterns of care and mortality of patients with ESRD in Sandy-affected areas (study group) and compare the results with the 2 comparison groups	7,791 (58.7%) patients in the study group received early dialysis. The percentage of participants who had ED visits was greater in the study group (4.1%) than in comparison group 1 (2.6%) and comparison group 2 (1.7%), both *P* < .001. The percentage of participants who were hospitalized during the week of the storm was greater in the study group than in comparison groups: 4.5% in study group, 3.2% in comparison group 1 (*P* < .001), and 3.8% in comparison group 2 (*P* = .003). 23% of study group participants who visited the ED received dialysis, compared with 9.3% in comparison group 1 (*P* < .001) and 6.3% in comparison group 2 (*P* < .001). Primary discharge diagnoses for patients visiting the ED or being hospitalized were for dialysis or ESRD. The 30-day mortality rate for patients in the study group (1.83%) was significantly higher than for comparison group 1 (1.47%; *P* < .001) and comparison group 2 (1.60%; *P* = .01).
Lin et al, 2014 (34)	Brooklyn, New York	Dialysis unit nurse managers (n = 15)	Retrospective survey conducted through interviews with a key focus on the influx of hemodialysis patients from closed dialysis centers to hospitals, coping strategies these hospitals used, and difficulties encountered	Determine the extent of surge of transient dialysis patients in hospital dialysis units from closed dialysis facilities during the storm and its aftermath, and explore difficulties encountered by hospitals in Brooklyn, New York in response to the patient surge	During and after Hurricane Sandy, 13 of 15 Brooklyn hospitals performed 347 hemodialysis sessions for transient hemodialysis patients. Influx of transient hemodialysis patients started before landfall, on October 28, 2012, rapidly increased after landfall, on October 29, 2012, and peaked on October 31, 2012. On peak day, dialysis units dialyzed 50.9% more patients than usual. Factors significantly associated with increased surge capacity were the average number of patients per day during nondisaster operations (*P* = .04), having affiliated outpatient dialysis centers (*P* = .03), use of extra dialysis machines (*P* = .01), and having extra staffing (*P* = .007). Storm-related challenges prevented the efficient operation of dialysis units; 7 of 14 operating hospital dialysis facilities reported a staff shortage due to transportation issues in getting to the facilities. All 5 affiliated outpatient dialysis centers cited communication challenges with ambulette service providers, which resulted in delays in transferring patients from EDs to outpatient dialysis centers. Closure of free-standing dialysis centers and other organizations presented communication challenges for hospital dialysis facilities.
Adalja et al, 2014 (35)	New York, New York	Health care professionals in clinical or administrative leadership roles (ie, nurses, EMS/hospital emergency management, administration) in departments likely to be affected by the increase in patient volume (N = 71)	Qualitative interview-based method: semi-structured open-ended questions addressed how the evacuations affected the facilities that received a large proportion of the evacuated patients	Examine the effect of the surge of dialysis patients on hospitals during Hurricane Sandy, describe operational challenges faced by these hospitals, and examine the coordination efforts among hospitals receiving patients	Communication challenges arose between receiving and evacuating hospitals. EMS teams’ unfamiliarity with the city’s geography and location of some receiving facilities presented challenges. Many hemodialysis patients who visited EDs for dialysis had missed ≥1 dialysis sessions, and some were in crisis. In some EDs, ED staff members corrected electrolyte imbalances until alternative dialysis arrangements could be made. One hospital anticipated the surge in dialysis patients, and as a result, it rapidly triaged dialysis patients.
**Hurricane Katrina**					
Kutner et al, 2009 (36)	New Orleans, Louisiana	Dialysis patients who were affiliated with clinics in the US Gulf Coast Katrina-affected area and the New Orleans metropolitan area (N = 5,031)	Retrospective cohort study using updated data from the United States Renal Data System Standard Analysis Files released in 2008	Investigate whether Hurricane Katrina’s landfall resulted in excess mortality among dialysis patients	Hurricane Katrina was not associated with excess mortality for dialysis patients in Katrina-affected areas (HR, 0.98; 95% CI, 0.86–1.11; *P* = .75) or among the subset of 2,238 dialysis patients who received treatment in the New Orleans area before the hurricane (HR, 0.90; 95% CI, 0.74–1.09; *P* = .28). Significant predictors of increased mortality were older patient age (HR, 1.03; 95% CI, 1.03–1.04; *P* < .001), Medicaid coverage (HR, 1.49; 95% CI, 1.34–1.66; *P* < .001) and hemodialysis as initial dialysis modality (HR, 1.96; 95% CI, 1.50–2.56; *P* < .001).
Anderson et al, 2009 (37)	New Orleans, Louisiana	Patients (N = 386) receiving dialysis at 9 New Orleans hemodialysis units	Cross-sectional survey: structured telephone interviews with questions addressing sociodemographic dialysis factors and evacuation characteristics	Estimate the percentage of New Orleans patients who missed hemodialysis sessions after Hurricane Katrina, and identify the factors associated with missed dialysis sessions and increased hospitalizations of hemodialysis patients post-Katrina	44% missed ≥1 dialysis session, and 16.8% missed ≥3 dialysis sessions post-Katrina. 8.6% of scheduled hemodialysis treatments were missed in the first month after the storm. Odds of missing ≥3 dialysis sessions, compared with missing no sessions, was 2.44 (95% CI, 1.14–5.24) for patients on dialysis for <2 years versus patients on dialysis ≥5 years. Patients who had <37 billed dialysis sessions (OR, 4.97; 95% CI, 1.57–15.8) and 37-38 billed sessions (OR 2.94; 95% CI, 1.11-7.80) were more likely to miss ≥3 dialysis sessions than patients who had ≥39 billing sessions in the 3 months before the storm. Patients who lived alone before the storm were more likely than patients who were cohabitating to miss ≥3 dialysis sessions (OR, 4.37; 95% CI, 1.85–10.3). 23% of participants reported being hospitalized in the first month after Katrina. Patients who missed ≥3 dialysis sessions were more likely to be hospitalized than patients who did not miss any sessions (OR, 2.16; 95% CI, 1.05–4.43).
Howard et al, 2012 (38)	Louisiana, Mississippi, Alabama	Patients from 103 clinics (outpatient and hospital-based) that had service disruptions during Hurricane Katrina (n = 5,861 hospitalized; n = 2,857 not hospitalized)	Retrospective observational study using data from the United States Renal Data System 2008 Standard Analytical Files	Estimate the impact of Hurricane Katrina on hospitalization rates among dialysis patients	Renal-related admissions rate for dialysis patients increased as a result of Hurricane Katrina, rising from 3.0 admissions per 100 patient-days in July 2004 to 5.5 admissions per 100 patient-days during September 2005. The rate ratio for renal-related hospitalizations associated with Hurricane Katrina was 2.53 (*P* < .001). The estimated number of excess renal-related hospital admissions attributable to Katrina was 140, roughly 3% of total dialysis patients at affected clinics.
Edmonson et al, 2013 (39)	New Orleans, Louisiana	Long-term hemodialysis patients receiving dialysis from 9 facilities in the New Orleans area 1 week before the landfall of Hurricane Katrina and were still alive 1 year later (n = 388)	Prospective cohort study	Determine the association of psychiatric symptoms (PTSD and depression), subsequent hospitalization, and mortality in the year after Hurricane Katrina among ESRD patients	92 (24%) reported symptoms consistent with a diagnosis of PTSD (posttraumatic stress disorder), and 178 (46%) reported symptoms consistent with a diagnosis of depression. 74 (19%) participants reported symptoms consistent with both PTSD and depression. 18 (5%) reported symptoms consistent with PTSD only, and 104 (27%) with depression only. Participants with depression, compared with participants without depression, were at a 33% higher risk of all-cause hospitalization and mortality (HR, 1.33; 95% CI, 1.06–1.66; *P* = .21) and cardiovascular-related (HR, 1.33; 95% CI, 1.01–1.76; *P* = .01) hospitalization and mortality. Participants with PTSD, compared with participants without PTSD, were not at significantly higher risk of all-cause hospitalization or mortality (HR, 1.11; 95% CI, 0.85–1.44; *P* = .23) or cardiovascular-related (HR, 1.14; 95% CI, 0.83–1.57; *P* = .21) hospitalization or mortality. However, participants with PTSD had a higher rate (not significant) of cardiovascular hospitalization and mortality.
**Hurricanes Katrina and Rita**					
Dossabhoy et al, 2015 (28)	Shreveport-Bossier, Louisiana	Dialysis patients visiting health care facilities in surrounding areas (notably Shreveport and Bossier, Louisiana) not directly affected by the hurricane (sample size not reported)	Narrative report: personal recollections and experiences of the authors	Describe the impact of hurricanes Katrina and Rita on the nephrology community, patients, and health care providers in areas not directly affected by the storm	Mass evacuation of hundreds of dialysis patients overwhelmed host hemodialysis centers; host hemodialysis centers compensated by providing up to 4 dialysis shifts per day at the time of maximum crisis. Surge of dialysis patients resulted in shortening dialysis treatments, which sometimes led to the development of uremic symptoms and inadequate dialysis. Arriving without knowledge of routine medication resulted in suboptimal treatment of comorbid conditions such as hypertension and diabetes. Closure of 2 of the 3 major transplant centers reduced the availability of cadaveric organs for transplantation and prolonged waiting times for patients on the transplant list.
**Mid-Atlantic storms**					
Abir et al, 2014 (33)	District of Columbia, West Virginia, Virginia, and Maryland	Charge nurse or supervisor in each dialysis facility (n = 81 of 90 centers approached)	Cross-sectional survey: semistructured interview guide. Survey questions addressed whether their centers lost power, and if so, duration of power loss, and where their patients received dialysis	Determine how large-scale power outages from the June 29, 2012, mid-Atlantic storms affected operations in a sample of hemodialysis centers in the affected regions	Of the 36 centers that lost power, 13 lost power for ≤12 hours; 9 lost power for 13–24 hours; 12 lost power for >24 hours, and 2 lost power for an unknown length of time. Of the 36 centers that lost power, 11 referred their patients to other dialysis centers, and 8 accommodated their patients during a later shift or on a different day. The power outage affected the operations of 24 dialysis centers. 8 centers that lost power received patients from other centers after restoration of their power, and 19 centers that were not affected by the power outage received patients from other centers. Some centers cited barriers in contacting patients by telephone to refer them to other centers as a result of the power outage. Respondents reported that despite making arrangements for their patients to receive treatment at alternate sites, some patients asked why they could not go to nearby EDs to receive dialysis, mentioning distance from home to alternate centers and transportation barriers.

Abbreviations: ED, emergency department; EMS, emergency medical services; ESRD, end-stage renal disease; HR, hazard ratio; ICD, *International Classification of Diseases*; IRR, incident rate ratio; OR, odds ratio; PTSD, posttraumatic stress disorder.

All 15 studies selected for full-text data extraction reported the effect of a hurricane or storm on dialysis patients (25–39). One study addressed Hurricane Maria (25), 8 addressed Hurricane Sandy (26,27,29–32,34,35), 5 addressed Hurricane Katrina (28,36–39), 1 addressed Hurricane Rita (28), and 1 addressed the June 2012 mid-Atlantic storms (33). All but 1 study was conducted in the continental United States (25), and only 1 study reported the effect on peritoneal dialysis patients (25).

### Indirect effects

Seven studies reported on the indirect effects of disasters on dialysis patients (25,28,30,33–35,37), including loss of electricity (25,33), lack of clean water (25), blocked roads (25), disruptions to the communication system (25), lack of transportation (34), mass evacuation and disturbed living situation (28,30), the surge of dialysis patients at hospitals (28,35), and missed dialysis sessions (37).

Loss of electricity and clean water can result in the closure of dialysis centers (25,33), which can lead to missed dialysis sessions, treatment delay (later in the day or next day), or referral to other centers (33). Another consequence was the development of bacterial peritonitis in 3 peritoneal dialysis patients who manually forced the fluid exchange (because of lack of electricity) or used river water (because of disrupted water supply) to clean the catheter exit site (25). Blocked roads and the lack of transportation presented challenges to transporting dialysis patients, and these challenges led to missed sessions (25,34).

Disruptions to living situations and the requirement for evacuation from residences can interrupt a dialysis patient’s usual source of care, which can place a strain on other centers as they face an increased patient load (28,30,35). Similarly, center closures and evacuation can have a ripple effect. When a center closes (or patients are evacuated), patients are shunted to another facility, where staff are forced to shorten treatments to meet the increased demand on units (28,35). When relocation is not an option, patients can miss 1 or more sessions, which can lead to electrolyte imbalances or ED visits (35). Closures can be complicated by disrupted communication, which can limit a center’s ability to communicate with patients or staff members about emergency plans (25).

Missed dialysis sessions among dialysis patients after a disaster were found to be associated with patients being on dialysis fewer than 2 years, living alone before the storm, and being unaware of the emergency plans of their dialysis center (37).

### Direct effects

Six studies reported on direct effects of disasters on dialysis patients (26,27,29,32,36,38) including increased ED use (26,27,29,32), number of hospitalizations (29,32,38), and mortality (32,36).

ED use and number of hospitalizations increased among dialysis patients in the week after the storm (26,27,29,32,38). However, effects on mortality were inconclusive. In 1 study, the 30-day mortality rate was higher among patients living in areas affected by a hurricane than either comparison group (32), whereas, in another study, the hurricane was not associated with excess mortality of dialysis patients (36).

### Mental health effects

Only 1 study addressed mental health among dialysis patients after a disaster (39). In a sample of patients with ESRD, after Hurricane Katrina, 92 (24%) reported symptoms consistent with a diagnosis of PTSD (posttraumatic stress disorder), and 178 (46%) reported symptoms consistent with a diagnosis of depression. Positive screening for depression was associated with higher risks for all-cause and cardiovascular-related hospitalization and mortality in the year after the storm (39).

### Other effects

Two studies reported on predisaster activities and their effects on dialysis patients postdisaster (30,31). Predisaster activities included dialysis-specific preparedness and early dialysis (receiving a session ahead of schedule). Dialysis-specific preparedness was associated with a significantly lower incidence of missed sessions (30). Similarly, receiving early dialysis was associated with a significantly smaller number of missed sessions (30) and reduced odds of 30-day mortality (31).

### Quality assessment

In our quality assessment (Appendix B), observational cohort studies met 5 to 8 of the possible 9 criteria of the Newcastle–Ottawa Scale. The criterion “outcome not present at start of study” was not met by any study because all studies assessed explored the exacerbation of an existing event (such as increases in ED use and hospitalization). The criterion “adequacy of follow-up of cohorts” was met by only 2 studies. Although no review complied with all 9 criteria assessed with the Newcastle–Ottawa Scale, after we converted the scale to good, fair, or poor quality categories, we determined that all but 1 study was of good quality.

We determined that 2 studies assessed by using the Critical Appraisal Skills Programme Qualitative Checklist were somewhat valuable, and 1 study was determined to be valuable. Additionally, the overall appraisal for all studies assessed by using the Joanna Briggs checklist was that these studies should be included in the review.

## Discussion

Findings from the 15 studies examined show that disasters have indirect, direct, mental health, and other effects on dialysis patients. The emergency preparedness recommendations identified in the study (Table 2) may be of use in an island setting, such as the Cayman Islands, because it has a 6-month–long hurricane season, and the health system is relatively small, making it difficult to deal with overflow from disasters. At the end of 2018, the Cayman Islands had 68 dialysis patients (K. Carol, email communication, November 6, 2018) and 2 dialysis units (one each in Grand Cayman and Cayman Brac). The dialysis unit in Grand Cayman can accommodate up to 11 dialysis patients at a time for an average of 33 dialysis sessions each day (40). With only 2 dialysis centers and 1 ED, patients are easily susceptible to treatment disruptions. Furthermore, key stakeholders (the director of public health, the deputy epidemiologist, and a nephrologist), expressed concerns about the growing dialysis population. Therefore, preparing to address the complex needs of ESRD patients is important.

**Table 2:** Emergency Planning Recommendations for Dialysis Patients

Identified Effects of Natural Hazards	Impact	Recommendations
**Indirect effects**		
Loss of electricity	Leads to closure of dialysis facilities and missed dialysis sessions.	Electricity and clean water are critical for dialysis; emergency planners could compensate for the loss of electricity by using generators and lack of clean water by making preparations to have extra storage of potable water; additionally, emergency planners and dialysis providers can make arrangements to transport patients to affiliate sites.
Lack of clean water	Leads to closure of dialysis facilities and missed dialysis sessions. Use of unclean water by peritoneal dialysis patients can lead to bacterial peritonitis	
Blocked roads and lack of transportation	Creates challenges in transporting dialysis patients and leads to missed dialysis sessions. Problems in the commute of staff members and providers to dialysis facilities can lead to a shortage of dialysis providers.	Emergency planners and dialysis centers should have a contingency plan to transport patients to another center; proactively evacuate dialysis patients living in vulnerable areas or those with limited mobility; make preparations for dialysis staff members and providers to shelter in place at dialysis units.
Disrupted communication system	Presents challenges in communicating with patients or staff members about emergency plans.	Develop an action plan of how to communicate with staff members ahead of disasters; provide dialysis patients with pertinent information before a hurricane, such as contact information for alternative dialysis centers, information on an emergency renal diet, copies of their dialysis orders, and a list of their medications and comorbidities.
Mass evacuation and disturbed living situation	Interrupts usual source of care for dialysis patients, leading to a strain on other centers as they face an influx of dialysis patients.	Identify dialysis patients from areas likely to experience mass evacuation and proactively admit these patients to the hospital, if possible; consider early dialysis and provide all dialysis patients with contact information for different dialysis centers to overcome surge problems.
Surge of dialysis patients at hospitals and dialysis units	Shortens treatment sessions for dialysis patients as dialysis centers grapple with trying to meet the increased demand on units.	Make plans to have dialysis providers readily available in alternate locations; have functioning dialysis centers open for extended hours and offer more treatment sessions to manage the increasing patient load.
Missed dialysis sessions	Leads to adverse health outcomes, such as visits to the emergency department, hospitalizations, and mortality.	Create and distribute a dialysis emergency packet, which should contain information for alternate dialysis locations; consider offering early dialysis
**Direct effects**		
Use of emergency department	Increase in emergency department visits for dialysis patients	Dialysis providers should consider offering early dialysis and provide dialysis patients with dialysis-specific preparedness knowledge, such as contact information for alternative sites, information on an emergency renal diet, copies of their dialysis orders, and a list of their medications and comorbidities.
Hospitalizations	Increase in hospitalizations for dialysis patients	
Mortality	—	
**Mental health effects**		
Posttraumatic stress disorder	Onset or exacerbation of posttraumatic stress disorder	In addition to preparing to manage the medical and social needs of dialysis patients after disasters, clinicians should prepare to screen dialysis patients for signs of depression, posttraumatic stress disorder, and other mental health conditions, and develop an action plan to address and treat the mental health needs of dialysis patients, such as referral to counseling and support groups.
Depression	Onset or exacerbation of depression	
**Others**		
Dialysis-specific preparedness	Lower the incidence of missed dialysis sessions	Periodically review dialysis-specific preparedness and awareness with dialysis patients, especially during the hurricane season; providers can assess the readiness of dialysis patients by using the disaster preparedness checklist provided by the National Kidney Foundation.
Early dialysis	Lower odds of missed dialysis sessions	Emergency planners should consider offering preemptive dialysis to curb adverse outcomes associated with missed dialysis sessions, such as emergency department visits and hospitalizations.

This review highlighted several implications for emergency planning in the island setting. The indirect effects of disasters — lack of electricity, clean water, and transportation; damage to communication systems; mass evacuation and disturbed living situations — resulted in the closure of dialysis centers, ESRD patient surge in host dialysis centers, missed dialysis sessions, difficulties communicating with providers and patients, and difficulties moving patients (25,28,30,33–35,37). These findings suggest that emergency preparedness planners and dialysis centers should have a contingency plan to transport patients to another center if dialysis units are rendered nonfunctional after storms. Lack of transportation, blocked roads, and dialysis patient surge are also significant concerns. Early evacuation can serve as a proactive approach for dialysis patients living in vulnerable areas and for those patients with limited mobility (25).

Similarly, having dialysis providers readily available in alternate locations and other plans to accommodate demand surges in host dialysis centers can help to address surge issues. Because the Cayman Islands has only 2 dialysis centers, managing patient surge is particularly important, because the closure of 1 dialysis center could likely overwhelm the remaining dialysis center. Emergency planners could prepare for this by keeping the functioning dialysis center open for extended hours to care for the increasing patient load and have dialysis providers readily available to address the needs of the dialysis community.

Another challenge is communicating with providers and patients when systems are disrupted. Information that is given to dialysis patients before a hurricane should include contact information for alternative dialysis centers, information on an emergency renal diet, copies of their dialysis orders, and a list of their medications and comorbidities (41). Providing patients with this information ahead of time can allow receiving centers to deliver care more efficiently to nonregular dialysis patients (28).

The direct effects of disasters include increases in ED use, hospitalizations, and mortality (26,27,29,32,36,38). Providing early dialysis in advance of a disaster is a proactive approach to curb these adverse outcomes. Receiving early dialysis was associated with lower odds of ED visits and hospitalizations in the week of the storm and reduced odds of 30-day mortality (31).

PTSD and depression symptoms are prevalent in the dialysis population post-disaster (39). Therefore, emergency planning for dialysis patients should include the identification and treatment of depression, PTSD, and other mental illnesses after disasters.

Our study has several limitations. The outcomes of interest were limited to the study’s definition of effects. Although we consulted with a research librarian to fully capture “effects” in our search, we may have missed terms that could have provided more value to our study. Also, only 1 study addressed the effects of disasters on peritoneal dialysis patients; therefore, findings may not be generalizable to this population. Most studies in this review addressed hurricanes; so, research exploring the effects of other types of disasters on dialysis patients is needed, particularly no-notice events such as earthquakes. Such disasters would preclude evacuation or opportunities for early dialysis. Publication and language bias are also possible limitations because we did not search the gray literature, and we included only articles in English. Finally, all but 1 study (25) reported findings in the continental United States. Dialysis patients living on islands may encounter additional challenges not present in nonisland settings.

Our study also has several strengths. We consulted with a research librarian; 2 reviewers independently searched the databases and screened the articles; and we searched 4 databases. These strengths helped to reduce selection bias and improve the scope of the studies included. Additionally, emergency preparedness recommendations are generalizable to other island settings with similar disasters.

Elucidating the effects of disasters on people whose lives depend on dialysis is of critical importance because the risk for adverse health outcomes increases when dialysis care is disrupted. The effects of disasters on dialysis patients have several implications for emergency planning. However, the topic is inadequately studied, especially in the island setting. The geographic isolation of islands can hamper the timely provision of resources to the dialysis community and presents a unique context to study the effects of disasters on dialysis patients.

Many islands have a tenuous health care system and lack economic safety nets, which can exacerbate the adverse outcomes of disrupted dialysis care. Efforts to mitigate the effect of disasters on dialysis patients will require coordination among public health professionals and other key personnel, carefully designed emergency preparedness plans, and education and training of all involved.

## Appendix A Electronic Search Terms Used in a Systematic Review of Natural Disasters in the Americas, Dialysis Patients, and Implications for Emergency Planning

**Table:**

Database	Electronic Search
PubMed	(((((((Disasters OR “Disasters”[Mesh] OR “Natural Disasters” OR “Natural Disasters”[Mesh] OR hurricanes OR Storms))) AND ((Dialysis OR “Dialysis”[Mesh] OR Renal Dialysis OR “Renal Dialysis”[Mesh] OR kidney failure OR “Kidney Failure, Chronic”[Mesh] OR renal failure OR hemodialysis OR peritoneal dialysis))) AND ((“Delivery of Health Care”[Mesh] OR healthcare delivery OR health-care delivery OR health care delivery OR Mortality OR “Mortality”[Mesh] OR “Morbidity”[Mesh] OR Morbidity OR morbidities OR “Hospitalization”[Mesh] OR hospitalization OR “emergency department use” OR “adverse outcomes” OR “Health Services Accessibility”[Mesh] OR “Quality of Life”[Mesh] OR “quality of life” OR “Patient Satisfaction”[Mesh] OR “Patient Care”[Mesh] OR “Patient Health Questionnaire”[Mesh] OR patient experiences OR “Patient Care Management”[Mesh] OR “Treatment Outcome”[Mesh] OR “Surveys and Questionnaires”[Mesh] OR “Health Care Surveys”[Mesh] OR complications OR “Mental Health”[Mesh] OR survey OR surveys OR Questionnaires OR Questionnaire)))))
Scopus	((TITLE-ABS-KEY (disaster*) OR TITLE-ABS-KEY ({natural disasters}) OR TITLE-ABS-KEY (hurricane*) OR TITLE-ABS-KEY (storm*))) AND ((TITLE-ABS-KEY (dialysis) OR TITLE-ABS-KEY ({renal dialysis}) OR TITLE-ABS-KEY ({kidney failure}) OR TITLE-ABS-KEY ({renal failure}) OR TITLE-ABS-KEY (hemodialysis) AND TITLE-ABS-KEY ({peritoneal dialysis}))) AND ((TITLE-ABS-KEY ({delivery of health care}) OR TITLE-ABS-KEY (healthcare W/2 delivery) OR TITLE-ABS-KEY ({health-care delivery}) OR TITLE-ABS-KEY (mortality*) OR TITLE-ABS-KEY (morbidity*) OR TITLE-ABS-KEY (hospitalization*) OR TITLE-ABS-KEY ({emergency department use}) OR TITLE-ABS-KEY ({adverse outcomes}) OR TITLE-ABS-KEY ({health services accessibility}) OR TITLE-ABS-KEY ({quality of life}) OR TITLE-ABS-KEY ({patient satisfaction}) OR TITLE-ABS-KEY ({patient care}) OR TITLE-ABS-KEY ({patient health questionnaire}) OR TITLE-ABS-KEY (patient W/2 experiences) OR TITLE-ABS-KEY ({patient management}) OR TITLE-ABS-KEY ({treatment outcome}) OR TITLE-ABS-KEY (survey*) OR TITLE-ABS-KEY (questionnaires*) OR TITLE-ABS-KEY ({health care surveys}) OR TITLE-ABS-KEY (complications*) OR TITLE-ABS-KEY ({mental health}))) AND (LIMIT-TO (PUBYEAR,2018) OR LIMIT-TO (PUBYEAR,2017) OR LIMIT-TO (PUBYEAR,2016) OR LIMIT-TO (PUBYEAR,2015) OR LIMIT-TO (PUBYEAR,2014) OR LIMIT-TO (PUBYEAR,2013) OR LIMIT-TO (PUBYEAR,2012) OR LIMIT-TO (PUBYEAR,2011) OR LIMIT-TO (PUBYEAR,2010) OR LIMIT-TO (PUBYEAR,2009)) AND (LIMIT-TO (LANGUAGE, “English”)
CINAHL	(MH “Disasters+” OR MH “Natural Disasters” OR disasters OR hurricanes OR Storms) AND (MH “Dialysis+” OR MH “Dialysis Patients” OR MH “Renal Replacement Therapy+” OR MH “Hemodialysis+” OR Renal Dialysis OR MH “Peritoneal Dialysis+” OR MH “Renal Insufficiency+” OR kidney failure OR renal failure OR hemodialysis OR dialysis OR peritoneal dialysis) AND (MH “Health Care Delivery+” OR healthcare delivery OR health-care delivery OR health care delivery OR MH “Mortality+” OR mortality OR MH “Morbidity+” OR morbidity OR morbidities OR MH “Hospitalization+” OR hospitalization OR MH “Emergency Care+” OR emergency department use OR adverse outcomes OR MH “Health Services Accessibility+” OR health services accessibility OR MH “Health Services Needs and Demand+” OR MH “Health Services+” OR MH “Treatment Outcomes+” OR MH “Quality of Life+” OR MH “Quality-Adjusted Life Years” OR MH “Quality of Working Life” OR MH “Psychological Well-Being+” OR quality of life OR MH “Patient Satisfaction+” OR patient satisfaction OR MH “Patient Care+” OR MH “Continuity of Patient Care+” OR patient care OR patient health questionnaire OR patient experiences OR patient care management OR MH “Treatment Outcome+” OR treatment outcome OR MH “Surveys+” OR surveys OR MH “Questionnaires+” OR questionnaires OR health care surveys OR complications OR MH “Mental Health” OR mental health)
Cochrane Library	A complete description of this search can be requested from the corresponding author.

## Appendix B Quality Assessment of Studies Included in Systematic Review of Natural Disasters in the Americas, Dialysis Patients, and Implications for Emergency Planning

**Table 1:** Quality Assessment Using the Newcastle–Ottawa Scale^a^ of Cohort Studies

Study	Selection (Maximum 1 ◆)				Comparab ility (Maximum 2 ◆)	Outcome (Maximum 1 ◆)			Quality
	Representativeness of Exposed Cohort	Selection of Nonexposed Cohort	Ascertainment of Exposure	Outcome Not Present at Start of Study		Assessment of Outcome	Length of Follow-Up	Adequacy of Follow-Up of Cohorts	
Malik et al (26)	◆	◆	◆		◆◆	◆	◆		Good
Lee et al (27)	◆	◆	◆		◆◆	◆	◆		Good
Gotanda et al (29)	◆	◆	◆		◆	◆	◆		Good
Lurie et al (31)	◆	◆	◆		◆◆	◆	◆		Good
Kelman et al (32)	◆	◆	◆			◆	◆		Poor
Kutner et al (36)	◆	◆	◆		◆◆	◆	◆	◆	Good
Howard et al (38)	◆	◆	◆		◆◆	◆	◆		Good
Edmonson et al (39)	◆	◆	◆		◆◆	◆	◆	◆	Good

^a^ Thresholds for converting the scale into good, fair, and poor quality are as follows: good, 3 or 4 diamonds in selection domain *and* 1 or 2 diamonds in comparability domain *and* 2 or 3 diamonds in outcome domain; fair, 2 diamonds in selection domain *and* 1 or 2 diamonds in comparability domain *and* 2 or 3 diamonds in outcome domain; poor, 0 or 1 diamond in selection domain *or* 0 diamonds in comparability domain *or* 0 or 1 diamonds in outcome domain. Source: Wells et al (20), Borge et al (23), Shurrab et al (24).

**Table 2:** Quality Assessment Using the Critical Appraisal Skills Programme Qualitative Checklist^a^

Criteria	Bonilla-Félix and Suárez-Rivera (25)	Dossabhoy et al (28)	Adalja et al (35)
**Section A: Are the results valid?**			
Was there a clear statement of the aims of the research?	Yes	Yes	Yes
Is a qualitative methodology appropriate?	Yes	Yes	Yes
Was the research design appropriate to address the aims of the research?	No	No	Can’t tell
Was the recruitment strategy appropriate to the aims of the research?	Can’t tell	Can’t tell	Yes
Was the data collected in a way that addressed the research issue?	Can’t tell	Yes	Yes
Has the relationship between the researcher and participants been adequately considered?	No	No	Can’t tell
**Section B: What are the results?**			
Have ethical issues been taken into consideration?	Can’t tell	Can’t tell	Yes
Was the data analysis sufficiently rigorous?	No	No	Can’t tell
Is there a clear statement of findings?	Yes	Yes	Yes
**Section C: Will the results help locally?**			
How valuable is the research?	Somewhat valuable: findings should be reviewed with caution because they may be heavily biased: study is based on personal recollections and experiences of the authors	Somewhat valuable: low-quality evidence; findings should be reviewed with caution because they may be heavily biased: study is based on personal recollections and experiences of the authors	Valuable: although findings should be reviewed with caution because no specific tool was used to group or organize identified themes

^a^ Options were yes, can’t tell, no. Source: Critical Appraisal Skills Programme (21).

**Table 3:** Quality Assessment Using the Joanna Briggs Checklist^a^ for Analytical Cross-Sectional Studies

Criteria	Murakami et al (30)	Abir et al (33)	Lin et al (34)	Anderson et al (37)
Were the criteria for inclusion in the sample clearly defined?	Yes	Yes	Yes	Yes
Were the study subjects and the setting described in detail?	Yes	Yes	Yes	Yes
Was the exposure measured in a valid and reliable way?	Unclear	Yes	Yes	Yes
Were objective, standard criteria used for measurement of the condition?	Unclear	Yes	Yes	Yes
Were confounding factors identified?	Yes	No	Yes	Yes
Were strategies to deal with confounding factors stated?	Yes	Not applicable	Yes	Yes
Were the outcomes measured in a valid and reliable way?	Unclear	Yes	Yes	Yes
Was appropriate statistical analysis used?	Yes	Not applicable	Yes	Yes
Overall appraisal	Include	Include	Include	Include

^a^ Options were yes, no, unclear, or not applicable. Source: Moola et al (22).
